# The Glycan Shield of HIV Is Predominantly Oligomannose Independently of Production System or Viral Clade

**DOI:** 10.1371/journal.pone.0023521

**Published:** 2011-08-16

**Authors:** Camille Bonomelli, Katie J. Doores, D. Cameron Dunlop, Victoria Thaney, Raymond A. Dwek, Dennis R. Burton, Max Crispin, Christopher N. Scanlan

**Affiliations:** 1 Department of Biochemistry, Oxford Glycobiology Institute, University of Oxford, Oxford, United Kingdom; 2 Department of Immunology and Microbial Science and IAVI Neutralizing Antibody Center, The Scripps Research Institute, La Jolla, California, United States of America; 3 Ragon Institute of MGH, MIT and Harvard, Boston, Massachusetts, United States of America; University of California , San Francisco, United States of America

## Abstract

The N-linked oligomannose glycans of HIV gp120 are a target for both microbicide and vaccine design. The extent of cross-clade conservation of HIV oligomannose glycans is therefore a critical consideration for the development of HIV prophylaxes. We measured the oligomannose content of virion-associated gp120 from primary virus from PBMCs for a range of viral isolates and showed cross-clade elevation (62–79%) of these glycans relative to recombinant, monomeric gp120 (∼30%). We also confirmed that pseudoviral production systems can give rise to notably elevated gp120 oligomannose levels (∼98%), compared to gp120 derived from a single-plasmid viral system using the HIV_LAI_ backbone (56%). This study highlights differences in glycosylation between virion-associated and recombinant gp120.

## Introduction

The functional envelope spike of HIV is a trimer of non-covalently associated gp120/gp41 heterodimers [1], densely coated with N-linked carbohydrates that are essential for correct glycoprotein folding and shielding vulnerable protein surfaces from antibody recognition [2], [3], [4], [5], [6], [7], [8], [9]. These carbohydrates are attached to the envelope proteins via the host cell glycosylation pathway [9], [10]. However, the glycosylation processing of virion-associated gp120 is divergent from that of typical glycoproteins produced by the host cell: the extensive array of gp120 N-linked glycans contains an ‘intrinsic’ patch of densely packed oligomannose glycans which are inefficiently trimmed by host ER and Golgi α-mannosidases [5], [11]. Such clusters of oligomannose-type carbohydrates do not occur in mammalian glycosylation and they therefore provide a potential target for selective antibody recognition of the virus [12]. Indeed, one of the few known broadly neutralising anti-HIV-1 antibodies, 2G12, exploits this divergence in host and viral glycan processing and recognises Manα1→2Man-linked residues attached to oligomannose termini within the gp120 ‘intrinsic’ mannose patch [12], [13], [14], [15], [16]. Along with other broadly neutralising antibodies, 2G12 confers sterilizing immunity to primary viral challenge in non-human primates [3], [17], [18], [19]. The Manα1→2Man array, recognised by 2G12, has become the blueprint for a range of microbial [15], [20], [21], [22], synthetic [16], [23], [24], [25] and recombinant glycoconjugate [26], [27] vaccine candidates against HIV-1. Additionally a number of lectins, specific for Manα1→2Man structures, exhibit potent antiviral activity [28], [29]. The abundance and conservation of Manα1→2Man motifs on the functional envelope of primary viral isolates is therefore crucial for the applicability of a carbohydrate-based vaccine approach and is the focus of this study.

Two recent studies have shown that α1→2-mannosidase trimming is reduced by the steric constraints imposed by gp120 trimerisation [11], [30] leading to a ‘trimer-associated’ oligomannose population in addition to the ‘intrinsic’ mannose patch. Both studies observed that, compared to recombinant gp120, there is a greater abundance of Manα1→2Man terminating structures (Man_6-9_GlcNAc_2_) on trimeric envelope glycoprotein. We previously described that Env, derived mostly from pseudoviral systems, was almost entirely oligomannose with a predominant population of Man_5_GlcNAc_2_ [11]. Here, we examine a wider range of viral production systems and envelope expression levels, and report a greater range of abundances of oligomannose-type glycans, although in all cases there is an elevation of oligomannose on virion-associated Env compared to recombinant, monomeric gp120.

## Results

**Figure 1:**
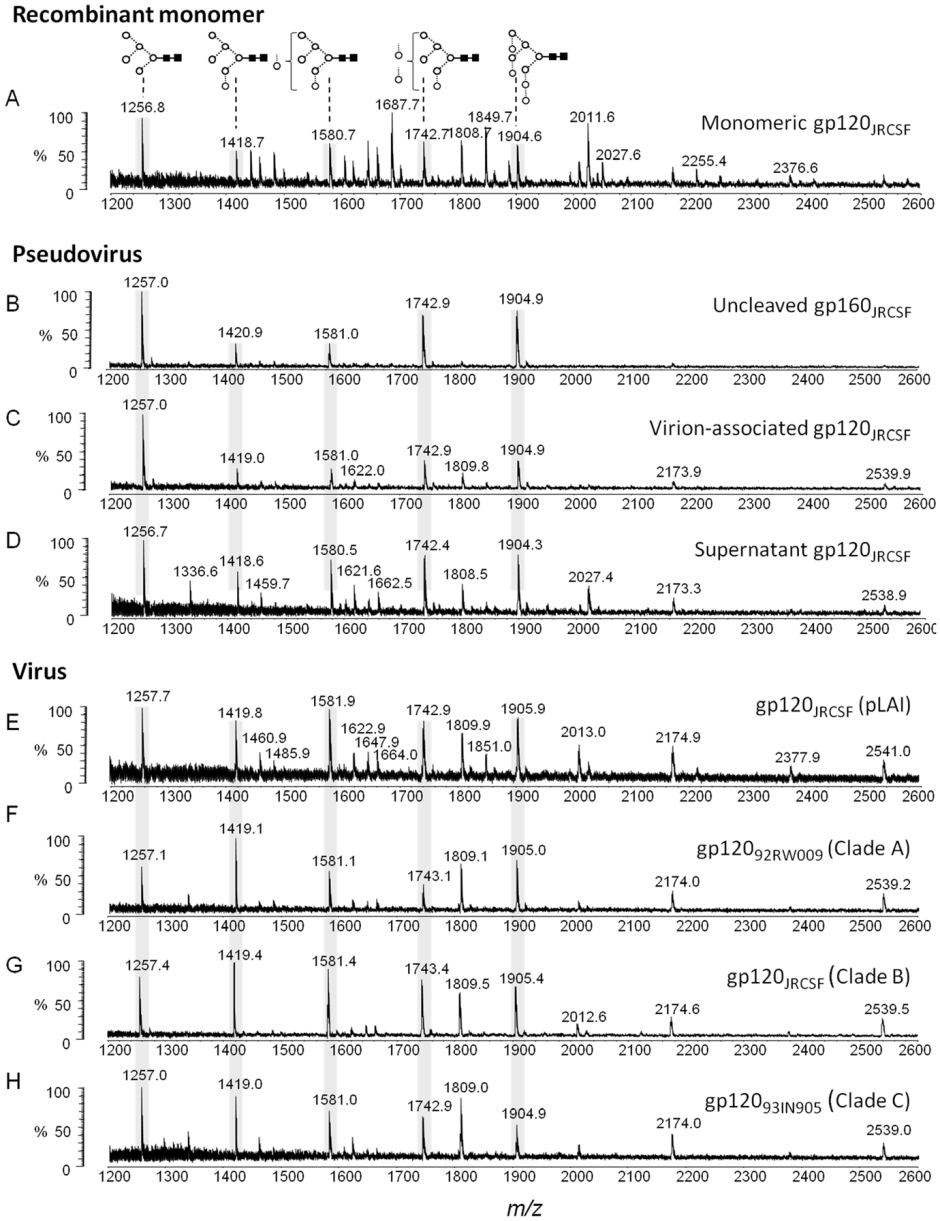
Comparison of recombinant, pseudoviral and viral gp120. MALDI-TOF MS analyses of released desialylated N-linked glycans ([M+Na]^+^ ions) from: (A) recombinant monomeric gp120_JRCSF_ expressed in HEK 293T cells; (B, C and D) respectively gp160_JRCSF_, gp120_JRCSF_ and soluble, non-virion associated envelope gp120_JRCSF_ isolated from pseudoviral particle preparations generated by transfection of HEK 293T cells with the pSVIII-JRCSF and pSG3Δenv plasmids at a ratio of 1∶10; (E) gp120_JRCSF_ isolated from replication competent viral particles generated by transfection of HEK 293T cells with pLAI-JRCSF env molecular clone; (F, G and H) respectively gp120_92RW009_, gp120_JRCSF_ and gp120_93IN905_ isolated from virus obtained by infection of human PBMCs. Symbols used for the structural formulae in this and subsequent figures: **⋄**  =  Gal, ▪  =  GlcNAc, ○  =  Man, 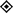  =  Fuc [46]. The linkage position is shown by the angle of the lines linking the sugar residues (vertical line  = 2-link, forward slash  = 3-link, horizontal line  = 4-link, back slash  = 6-link). Anomericity is indicated by full lines for β-bonds and broken lines for α-bonds [46]. The oligomannose series are highlighted.

As previously reported, the matrix-assisted laser desorption/ionisation time of flight (MALDI-TOF) mass spectrometry (MS) spectrum for recombinant wild-type gp120_JRCSF_ showed extensive complex-type glycosylation [11], with the intrinsic mannose patch forming around 29% of the total glycan population (Figure 1A). The abundances of oligomannose- and complex-type N-linked glycans released from gp120 in this and subsequent production systems are shown in Table 1.

**Table 1:** Abundances of released N-linked glycans obtained from recombinant (monomeric), pseudoviral, and viral gp120†.

gp120 source	Cell-type	Man_5-9_GlcNAc_2_%	Man_5_GlcNAc_2_%	Complex%	Mannose content relative to rgp120‡
Recombinant monomer (pHLsec JRCSF)	293T	29%	7.7%	71%	1.0
Pseudovirus (pSG3Δenv:pSVIII JRCSF, 2∶1)	293T	98%	38%	2%	3.4
Pseudovirus (pSG3Δenv:pSVIII JRCSF, 10∶1)	293T	85%	39%	15%	2.9
Supernatant (pSG3Δenv:pSVIII JRCSF, 10∶1)	293T	73%	18%	27%	2.5
Virus (pLAI-JRCSF env)	293T	56%	10%	44%	1.9
Virus JRCSF (clade B)	PBMC	79%	12%	21%	2.7
Virus 92RW009 (clade A)	PBMC	64%	10%	36%	2.2
Virus 93IN905 (clade C)	PBMC	62%	19%	38%	2.1

†Abundances obtained for desialylated N-linked glycans released from gp120 described in this study. Values were obtained from data presented in Figure 1 and Doores *et al.* [11].

‡Values represent the increase in oligomannose population (Man_5-9_GlNAc_2_) for pseudoviral and viral gp120 compared to monomeric, recombinant gp120.

Pseudoviral particles were prepared using human embryonic kidney (HEK) 293T cells with plasmids carrying JRCSF envelope gene (pSVIII-JRCSF) and the HIV-1 backbone (pSG3Δenv) at a ratio of 1∶10 respectively. A recent study by Crooks *et al.* has shown that pseudoviral production systems produce significant levels of non-functional uncleaved ‘gp160ER’ whose glycans are entirely sensitive to digestion by endoglycosidase H (endo H) [31]. In addition to ‘gp160ER’, a smaller population of partially endo H-resistant cleaved gp120/gp41 trimers (indicative of the presence of some complex-type glycans) was observed and it was proposed that only this more processed glycoform is shed from the functional envelope spike into the supernatant. We used mass spectrometry to determine the divergent glycosylation of these two species and showed that both gp160, and the less abundant virion-associated gp120, consisted predominantly of oligomannose glycans (94% and 85% respectively) (Figure 1B, C). Interestingly, increasing the Env:backbone plasmids ratio from 1∶10 to 1∶2 (constant DNA) resulted in an increased level of envelope expression [32] (data not shown) and an even higher oligomannose abundance (>98%, as previously reported [11]) suggesting envelope expression level might influence the glycosylation profile of gp120. We observed an unusual abundance of Man_5_GlcNAc_2_, indicating that most of the virion-associated material had not been exposed to the medial-Golgi-resident GlcNAc transferase I (GnT I). This lack of processing is also consistent with the abundance of uncleaved gp160, in this pseudoviral systems: the furin protease, responsible for gp160 cleavage into gp120/gp41, is proposed to be largely resident in the trans-Golgi apparatus [33], [34].

In contrast to virion-associated gp160/120, the gp120 shed into the supernatant, proposed to derive solely from cleaved functional trimers [31], contained more complex-type glycans (27%) (Figure 1D) but was nonetheless mostly oligomannose (73%). This elevated level of oligomannose glycans compared to recombinant monomeric gp120 (Figure 1A) is consistent with the reduced mannose trimming previously reported for recombinant, trimeric gp120 compared to recombinant, monomeric gp120 [11]. Moreover, the 27% complex-type glycans seen in this shed gp120 was matched by a corresponding reduction in the Man_5_GlcNAc_2_ peak compared to virion associated gp120 (Figure 1C) indicating this species does not evade processing by GnT I and subsequent Golgi-resident glycosidases and glycosyltranferases.

We next compared the glycosylation of pseudovirus-derived gp120 to replication competent virus-derived gp120. The glycans from gp120 derived from JRCSF virus prepared in HEK 293T cells using an infectious pLAI-JRCSF Env molecular clone [35] showed a more even division between oligomannose (56%) and complex-type glycans (44%), and a more equal distribution of abundances within the Man_5–9_GlcNAc_2_ structures (Figure 1E). The complex-type glycans were predominantly of the bi- or tri-antennary type with variable galactosylation and fucosylation typical for HEK 293T cells [36], [37]. We observed a reduced envelope expression level in these replication competent viral particles compared to the pseudoviral particles. This reduced envelope expression level and corresponding reduction in oligomannose abundance further suggests envelope expression levels may influence the glycosylation profile of virion-associated gp120. In addition to cleaved gp120, uncleaved, non-functional gp160 was also detected in the pLAI-JRCSF Env virus derived membrane-associated fraction. The analysis of gp160 glycosylation revealed, as for the pseudoviral derived gp160, less efficient processing by the Golgi α-mannosidases IA–C, with elevated populations of Manα1→2Man linked oligomannose glycans compared to gp120 (68% Man_6–9_GlcNAc_2_ for gp160 compared to 46% Man_6–9_GlcNAc_2_ for gp120; data not shown). This suggests that uncleaved gp160 adopts a quaternary arrangement with more occluded glycans compared to cleaved gp120/gp41.

Analyses of gp120 derived from virus prepared by infection of peripheral blood mononuclear cells (PBMCs) with viruses from clade A (92RW009), clade B (JRCSF), and clade C (93IN905) showed a predominantly oligomannose glycan composition (62–79% Man_5–9_GlcNAc_2_, Figure 1F, G, H) with a distribution similar to that previously reported for PBMC-derived gp120_JRCSF_ [11]. In a previous study we noted the presence of some complex-type glycans but due to limitations of material we were unable to perform analysis of desialylated material required to distinguish these glycans from those of the capture antibodies [11]. Here, MALDI-TOF MS analysis of desialylated glycans revealed, in addition to the Man_5–9_GlcNAc_2_ glycans, a smaller series of branched, fucosylated complex-type glycans at *m/z* 1809 (11–24%), 2012 (1.5–3%), 2174 (3–5.5%) and 2539 (2.5–4.6%) in all three spectra corresponding to the neutral derivatives of sialylated bi, tri and tetra-antennary glycans.

Overall, the glycan distribution within the oligomannose series is similar to that observed for the single-plasmid infectious pLAI-JRCSF env clone (Figure 1E) and the shed material from the pseudoviral system (Figure 1D), with some complex-type glycans and without an elevated Man_5_GlcNAc_2_ peak. We note however that the distribution of the oligomannose series differs slightly between isolates: the ratio of oligomannose-type glycans that terminate with Manα1→2Man, compared to those that do not, is higher for 92RW009 (clade A, 5.2) and JRCSF (clade B, 5.6) than for 93IN905 (clade C, 2.7). A likely explanation for this difference in glycan processing, in clade C envelope, is the absence of key glycosylation site(s) which reduce the density of the intrinsic mannose patch and increase the processing of adjacent Manα1→2Man termini. Notably, the oligomannose glycan attached to Asn295 is absent in most clade C isolates, including HIV-1 93IN905, and is critical for efficient neutralisation by a number of mannose-specific ligands, including 2G12 [38].

Therefore, as for pseudoviral and viral particles obtained from HEK 293T cells (Figure 1C, E), the glycans on PBMC-derived virus from isolates from distinct antigenic and geographical backgrounds are predominantly oligomannose.

## Discussion

**Figure 2:**
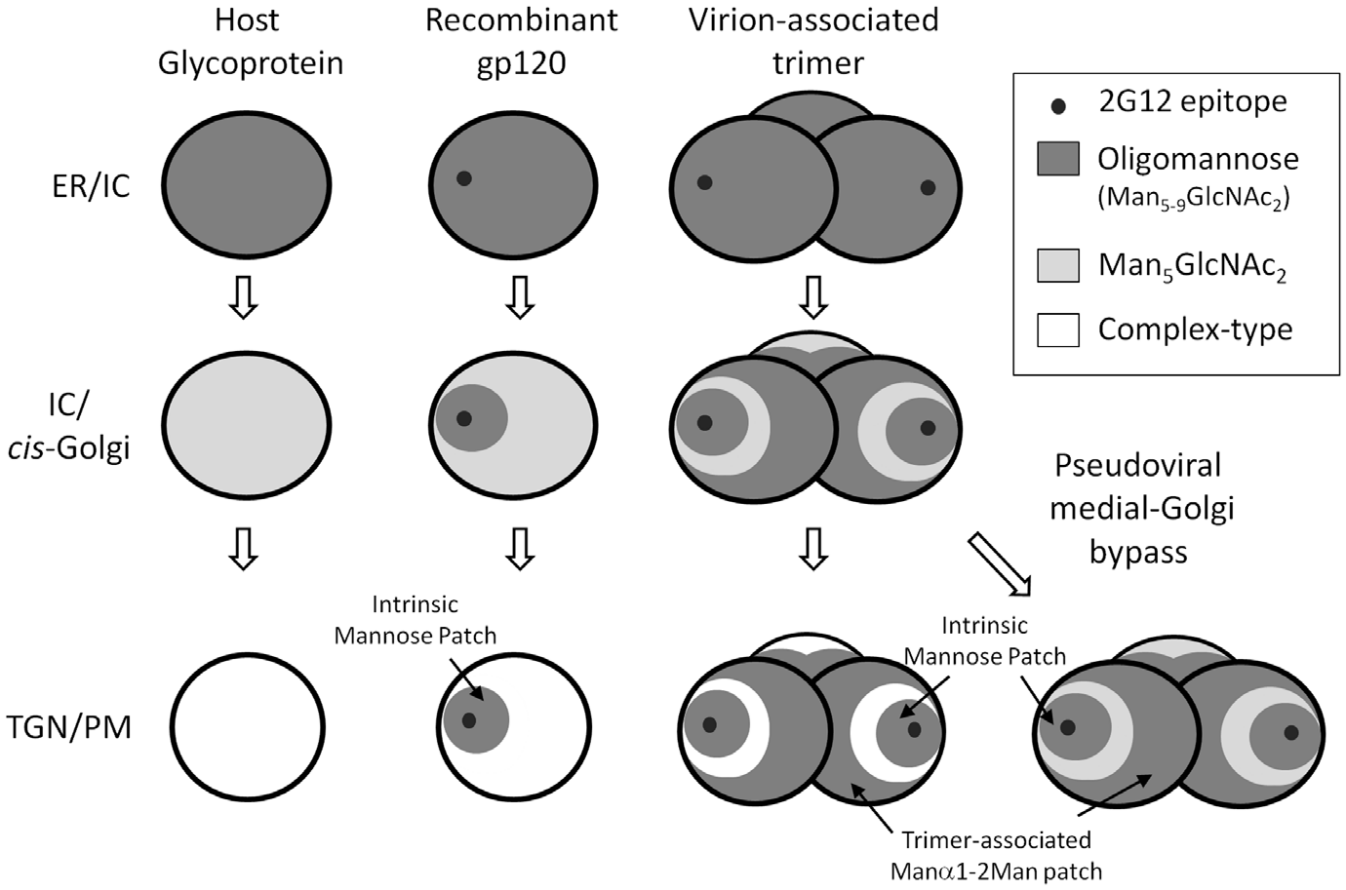
Multiple divergences of gp120 glycosylation from host cell glycosylation. Following removal of terminal α-linked glucose residues in the ER, folded glycoproteins contain exclusively oligomannose glycans. During transit through the ER, intermediate compartment (IC) and *cis*-Golgi apparatus, Manα1→2Man termini are removed by ER Mannosidase I and Golgi Mannosidases A–C to yield Man_5_GlcNAc_2_. However, the oligomannose cluster intrinsic to monomeric gp120 [5], [14] limits glycan processing on both monomeric and oligomeric gp120 [11], [30]. The steric consequences of trimerisation further limit Manα1→2Man trimming [30] leading to an additional ‘trimer-associated’ population of Man_5–9_GlcNAc_2_. The exposed Man_5_GlcNAc_2_ glycans on gp120 that passage through the full extent of the Golgi apparatus and *trans* Golgi network (TGN) to the plasma membrane (PM) are processed by GnT I and subsequent enzymes to form complex-type glycans. However, envelope glycoprotein that does not follow this route to the PM is characterized by an elevated abundance of Man_5_GlcNAc_2_ (and closely resembles gp120 expressed in GnT I-deficient cells [11], [30]), and reduced furin cleavage. Thus the intrinsic mannose patch, which includes the 2G12 epitope, persists from the earliest stages of glycan processing whilst other elements of the glycan shield exhibit variably processed glycans depending on oligomerization state and, at least in the case of pseudoviral gp160/gp120, cellular trafficking.

The HIV envelope is entirely processed by the glycosylation machinery of the host cell: the interaction of envelope with the spectrum of enzymatic activities present in the secretory pathway determines the types of glycans that will be presented on gp120 at the virion surface or as a recombinant protein. Although all N-linked glycosylation sites on gp120 are initially glycosylated with the same Glc_3_Man_9_GlcNAc_2_ precursor, these sites are not processed equivalently. We propose a model (Figure 2), based on the data reported here and integrating previous findings from our group and others, of how gp120 is processed as it traffics through the cell. First, the ER glycoform arises following the removal of the final glucose residue by α-glucosidase II to produce Man_9_GlcNAc_2_ (or depending on the cell type by the action of endomannosidase to yield D2,D3-Man_8_GlcNAc_2_). This natural gp120 glycoform, normally a transient biosynthetic intermediate, has been isolated in a number of studies using inhibitors of α-mannosidases such as kifunensine [14], [27], [39]. The Man_8–9_GlcNAc_2_ intermediates are then processed by the ER and Golgi α-mannosidases. This process is slower for glycans within the intrinsic mannose patch, and is further limited by the steric consequences of trimerisation [11], [30] (Figure 1 and 2). These two factors combine to yield an enhanced abundance of Manα1→2Man terminating glycans compared to recombinant monomeric gp120 which is largely insensitive to changes in expression system or envelope structure. Finally, the more exposed regions of gp120 are processed by the medial Golgi resident GnT I to form the hybrid-type glycan, GlcNAcβ1→2Man_5_GlcNAc_2_, and subsequent complex-type glycosylation found on cell surface and on virions. These complex-type N-glycans are processed in a tissue-specific manner, consistent with observations that they are not essential for viral function but may modulate infectivity and accessibility of some antibody epitopes [40]. The predominance of the biosynthetic intermediate, Man_5_GlcNAc_2_ (and the absence of complex-type glycans), and reduced gp160 processing are both markers for a lack of processing in the medial-Golgi apparatus. Both these phenomena are observed in envelope glycoproteins isolated from pseudoviral particles, and the mechanism for this Golgi by-pass, which is consistent with a recent study showing an abundance of ‘gp160ER’ on pseudoviral particles [31] is unknown, but might reasonably be attributed to either an alteration of compartmentalisation or to a substrate saturation of Golgi-resident envelope processing enzymes.

Overall, the data presented here and in our previous study [11] indicate that the glycosylation of HIV envelope glycoproteins diverges from typical host-cell glycosylation on at least three levels. First, the clustering of N-glycans gives rise to an ‘intrinsic’ mannose patch (Figure 1A). Second, the steric constraints of trimerisation result in an additional population of oligomannose glycans. Third, in pseudoviral systems, a majority of envelope glycoproteins bypass the Golgi-resident enzymes responsible for complex glycan biosynthesis and protein cleavage, leading to an unusual elevation of Man_5_GlcNAc_2_ on non-functional envelope gp160. The ‘intrinsic’ and ‘trimer-associated’ mannose patches give rise to a predominance of oligomannose-type glycans on virion-associated gp120 that is conserved regardless of virus production system, envelope expression level, 2G12 sensitivity or envelope sequence.

## Materials and Methods

### Ethics statement

Human blood samples from healthy donors were obtained from The Normal Blood Donor service at The Scripps Research Institute. The collection of human blood samples for isolation of PBMCs and subsequent propagation of HIV-1 virus was approved by the Institutional Review Board at The Scripps Research Institute (protocol number HSC-06-4604).

### Recombinant protein expression

HEK 293T (ATCC number CRL-1573) were cultured in Dulbecco's Modified Eagle Medium (DMEM) supplemented with 10% fetal calf serum, penicillin and streptomycin. Transient transfection using the pHLsec vector followed that of Aricescu [41]. Briefly, for each T175 flask, 90 µg of polyethyleneimine (PEI) and 50 µg of DNA were incubated for 10 min in 5 mL of serum free media; then added to 80–90% confluent cells cultured in 25 mL of serum free media. Culture supernatant was collected at 4 days post-transfection, and subsequently centrifuged, sterile filtered and then concentrated by centrifugal filtration using Vivaspin 20 devices.

### Pseudovirus and virus preparation in 293T cells

Pseudovirus was generated in HEK 293T cells as described [42]. Briefly, HEK 293T cells were transfected with plasmids carrying the reporter gene expressing the virus backbone (pSG3Δenv) and the functional envelope clone (pSVIII-JRCSF) at a ratio of 2∶1 or 10∶1 (total DNA, 60 µg per 7×10^6^ cells) using Fugene (Roche) according to the manufacturer's instructions. Virus supernatants were harvested after 3 days. Fully replicative JRCSF virus capable of multiple round infection was made in 293T cells by transfection with a single plasmid construct (pLAI-JRCSF env) using Fugene [35].

### Virus preparation in PBMCs

Human PBMCs were obtained from healthy individuals and isolated and stimulated as previously described [43]. HIV-1_JRCSF_, HIV-1_92RW009_ and HIV-1_93IN905_ virus stocks were grown and titered on CD8^+^-depleted PBMCs [44]. Virus production was monitored by p24 ELISA (Aalto Bioreagents, Dublin, Eire).

### Envelope Isolation

Virus preparations were pre-cleared by low speed centrifugation. Virus particles were pelleted by ultracentrifugation (22,000 rpm, 1 hour). Virus pellets were lysed with NP-40 (1% in PBS with protease inhibitors, 20 mins at 4°C). The debris was removed by centrifugation and the envelope protein was immunoprecipitated with HIV envelope specific monoclonal antibodies (D7324, b12, b6, F425-b4e8, VRC01, VRC03, PGV04) depending on virus isolate). Protein A and G beads were added and incubated overnight at 4°C. The beads were washed 5 times with PBS and then the protein was eluted by heating in loading buffer (containing dithiothreitol) for 10 mins at 100°C and resolved by SDS-PAGE. The envelope band was confirmed by western blot (primary antibodies; 2G12, F425-b4e8, PGV04, HIVIG (depending on strain), secondary antibody, goat-anti-human-Fcγ-HRP) and cut to use directly in glycan analysis. The ‘soluble non-virion associated fraction’ is the envelope protein isolated by immunoprecipation of the supernatant after the virus has been removed by ultracentrifugation.

### MALDI-TOF mass spectrometry

Oligosaccharides were released from target glycoproteins with Peptide-*N*-Glycosidase (PNGase) F (New Englands Biolabs) from Coomassie blue-stained NuPAGE [45]. Excised bands were washed five times alternatively with acetonitrile and deionised water, and rehydrated with a 3000 Units/ml of PNGase F water solution. After incubation for 12 hours at 37°C, the enzymatically released N-linked glycans were eluted with water. Samples were analysed by positive ion matrix-assisted laser desorption/ionization (MALDI) time-of-flight (TOF) mass spectra with a Shimazu AXIMA TOF^2^ MALDI TOF/TOF mass spectrometer (Kratos Analytical, Manchester, UK) fitted with delayed extraction and a nitrogen laser (337 nm). Samples were cleaned on a Nafion 117 membrane (Aldrich), and then prepared for mass spectrometry by adding 0.5 µL of an aqueous solution of the glycans to the matrix solution (0.3 µL of a solution of 2,5-dihydroxybenzoic acid in acetonitrile:water (1∶1, v:v) on the stainless steel target plate and allowing it to dry at room temperature. The sample/matrix mixture was then recrystallized from ethanol. Samples were examined after removal of any potential sialic acids by heating at 80°C for 1 hr with 1% acetic acid.
